# A transcriptomics-based meta-analysis identifies a cross-tissue signature for sarcoidosis

**DOI:** 10.3389/fmed.2022.960266

**Published:** 2022-09-20

**Authors:** Yale Jiang, Dingyuan Jiang, Ulrich Costabel, Huaping Dai, Chen Wang

**Affiliations:** ^1^Department of Pulmonary and Critical Care Medicine, China-Japan Friendship Hospital, Beijing, China; ^2^Clinical Trial Center, National Cancer Center/National Clinical Research Center for Cancer/Cancer Hospital Chinese Academy of Medical Sciences, Peking Union Medical College, Beijing, China; ^3^School of Medicine, Tsinghua University, Beijing, China; ^4^National Center for Respiratory Medicine, Institute of Respiratory Medicine, National Clinical Research Center for Respiratory Disease, Chinese Academy of Medical Sciences, Beijing, China; ^5^Department of Pneumology, Center for Interstitial and Rare Lung Diseases, Ruhrlandklinik, University Hospital, Essen, Germany; ^6^Tsinghua University-Peking University Joint Center for Life Sciences, Beijing, China; ^7^Peking Union Medical College, Beijing, China

**Keywords:** sarcoidosis, transcriptome, interferon, IL-17, machine learning

## Abstract

Sarcoidosis is a granulomatous disease of unknown etiology, immunologically characterized by a Th1 immune response. Transcriptome-wide expression studies in various types of sarcoid tissues contributed to better understanding of disease mechanisms. We performed a systematic database search on Gene Expression Omnibus (GEO) and utilized transcriptomic data from blood and sarcoidosis-affected tissues in a meta-analysis to identify a cross-tissue, cross-platform signature. Datasets were further separated into training and testing sets for development of a diagnostic classifier for sarcoidosis. A total of 690 differentially expressed genes were identified in the analysis among various tissues. 29 of the genes were robustly associated with sarcoidosis in the meta-analysis both in blood and in lung-associated tissues. Top genes included *LINC01278* (*P* = 3.11 × 10^–13^), *GBP5* (*P* = 5.56 × 10^–07^), and *PSMB9* (*P* = 1.11 × 10^–06^). Pathway enrichment analysis revealed activated IFN-γ, IL-1, and IL-18, autophagy, and viral infection response. IL-17 was observed to be enriched in peripheral blood specific signature genes. A 16-gene classifier achieved excellent performance in the independent validation data (AUC 0.711–0.964). This study provides a cross-tissue meta-analysis for expression profiles of sarcoidosis and identifies a diagnostic classifier that potentially can complement more invasive procedures.

## Introduction

Sarcoidosis is a systemic disorder featured by the presence of non-caseating granuloma. The incidence varies considerably depending on sex, age, ethnicity and geographic regions, indicating that both genetic predisposition, and environmental factors play essential roles in the pathogenesis. The etiology of sarcoidosis remains uncertain despite decades of effort. Multiple genome-wide expression studies have been performed on sarcoidosis in order to understand underlying molecular mechanisms, including directly affected tissues such as lung and skin, fluids in contact with granulomas like bronchoalveolar lavage (BAL), and peripheral blood.

Expression profiles of circulatory blood and sarcoid tissues are quite different, but can be implicated in pathways involved in innate and adaptive immunity, granuloma formation, and fibroproliferation (1–3). Th1 associated molecules, especially INF-γ response transcription factor STAT1 as well as STAT1 regulated chemokines (IL-5, IL-7, IL-15, CCR5, CXCL9, CXCL10, and CXCL11) have been recognized as key inflammatory factors in sarcoidosis in transcriptome-wide analysis of lung, lymph nodes and peripheral blood (4, 5).

Aside from tissue-independent common pathways, genes enriched in IFN signaling (type I and II) and the Th17 pathway, including *IL-23*, *IL-23R*, and *IL-21*, are dysregulated in skin tissue of active cutaneous sarcoidosis (6). Upregulated genes in orbital tissues further validated the role of IFN-γ and type I IFNs, including *CXCL10, GBP5, STAT1, AIM2, ICAM1*, and *JAK2* (7). Enrichment analysis of transcription factor binding sites revealed that interferon-response factor 1 and 2 (IRF-1 and IRF-2), and nuclear factor κB, are involved in the transcriptional modulation in sarcoidosis.

In addition to pathways associated with adaptive immune system and T-cell signaling, differentially expressed genes in BAL identified a novel gene network linkage between immunoproteasome subunits (*PSMB-8, −9, −10*), and found *TWIST1*, a biomarker of M1-activation, to be up-regulated in sarcoidosis patients compared to controls (8, 9). Comparison of BAL cells from patients with severe and stable sarcoidosis demonstrated increased expression of protein kinase *TYK2* and cell cycle inhibitor *p21Waf1/Cip1*, as well as reduced expression of Cathelicidin (CAMP), confirming the involvement of Th1 and INF-γ immune responses (10, 11). *MMP12* and *ADAMDEC1* were newly identified to be significantly associated with sarcoidosis severity in lung tissue and BAL (5).

Peripheral blood has also been extensively examined for sarcoidosis specific gene identification. Whole blood gene expression signature distinguished sarcoidosis from healthy controls with an error rate of 12.1% (12). The genes belonged to Th1-type inflammation, such as INF signaling pathway (*IFN*, *STAT1*), and to T-cell homeostasis and survival (*IL-15* and *IL-7R*). A 20 gene signature was identified in peripheral blood mononuclear cells (PBMC) with an accuracy of 86.0% to distinguish healthy subjects from those with sarcoidosis, but performed less well when applied to replication datasets (13). Unlike the prior model, the cohort-specific 20 gene signature was not composed of genes belonging to T-cell, JAK/STAT, or cytokines pathways.

The diagnosis of sarcoidosis relies on histopathologic examination and compatible clinical presentation, with other causes of granulomatous inflammation excluded (14). A definitive diagnosis is challenging because several other diseases can show similar histopathologic changes (15). The prognosis is less favorable among patients with more advanced stage at diagnosis, emphasizing the significance to develop auxiliary approaches to help early diagnosis of such potentially hazardous disease. However, the clinical value of sarcoid tissue-based diagnostic gene markers remains unclear owing to the limitations of obtaining biopsy samples.

In this study, a full meta-analysis was performed utilizing all available genome-wide expression datasets for sarcoidosis vs. healthy subjects from public database to explore robust gene markers across different tissues and to propose an expression-oriented diagnostic panel. To our knowledge, this is to date the largest cross-tissue transcriptomic meta-analysis of sarcoidosis.

## Materials and methods

### Dataset identification

**FIGURE 1:**
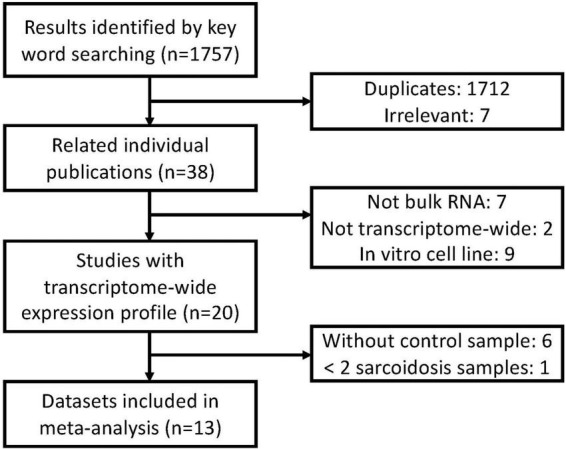
Overview of the meta-analysis approach for development and validation of sarcoidosis transcriptome panel.

A systematic database search was performed on Gene Expression Omnibus (GEO). A total of 1,757 records were found with sarcoidosis as search term and organism confined to Homo sapiens (Feb 5 2021). We excluded 1,712 duplicated and 7 irrelevant results. Nine cell lines were excluded since they do not depict transcriptional features and functions *in vivo*. 3 methylation datasets, 1 single-cell dataset, 3 non-coding RNA datasets, 1 dataset with less than two sarcoidosis subjects, and 6 datasets without compatible control were subsequently excluded. Two array-based studies without transcriptome-wide data were removed, and 13 datasets were finally included in our meta-analysis (Figure 1). We checked definition of sarcoidosis and control in each of the included study. Diagnosis of sarcoidosis was made by a sarcoidosis specialist, biopsy evidence, compatible clinical, and radiological findings according to the WASOG guidelines (15) in GSE19314, GSE42834, GSE16538, and GSE37912. Similar diagnosis criteria were applied in GSE83456 and GSE75023. The other studies used pathology-confirmed biopsy displaying typical non-necrotizing epithelioid granuloma as definition. Controls were defined as recruited healthy volunteers or disease-uninvolved tissues.

### Quality control and pre-processing

Selected datasets downloaded from the GEO repository^1^ consist of different forms of expression measurements and probe annotation files. R package limma and affy were utilized to concordantly process the datasets to enable comparison (16, 17). For datasets with available raw CEL files for download, expression intensities were extracted and normalized using robust multi-array average (RMA) based on the corresponding custom chip definition file (CDF). Only genes estimated to be present in more than 10% samples were included in subsequent analysis. Data generated from Affymetrix HGU133 Plus 2.0, Affymetrix Human Gene/Exon 1.0 ST Array, and Illumina HumanHT-12 V4.0 expression beadchip underwent procedures described above. For RNA sequencing data generated from Illumina HiSeq 3000 platform, raw counts were downloaded and normalization factors were calculated with edgeR (18). Probes of different arrays were subsequently replaced by official gene symbols, and multiple expression measurements were collapsed by maximum value when one gene has replicative measurements.

### Differential gene expression analysis for generating meta-signature

We performed differential expression analysis on individual datasets by comparing sarcoidosis vs. normal samples using a linear model-based R package LIMMA (16). Considering limited and incomplete demographic and clinical variables available in public database, we used permutated unwanted variation estimation instead of including available but incomplete variables into the model to test difference between cases and controls. To identify, estimate and remove unwanted sources of variation to compensate for incomplete information of samples provided by public datasets, surrogate variable analysis was applied to each dataset using the “leek” method (19). The estimated surrogate variables were used as covariates in the formula of differential expression analysis. The probes with Benjamini-Hochberg corrected *P*-value < 0.05, with multiple-testing adjusted, were considered as significant (20). Since inconsistence in terms of study design, cohorts, measurements, etc., meta-analysis was performed with metafor package using residual maximum likelihood (REML) model (21). We performed the analysis in blood sets, lung-associated sets, and all available sets, respectively. Pathway enrichment analysis was further conducted with ClueGO based on GO biological process databases (08/05/2020) (22). For differentially expressed genes (DEGs) in the meta-analysis of blood and lung-associated tissues, significant genes in one meta-analysis but not in the other were identified as tissue-specific. Top genes of these tissue-specific genes were also enriched for biological processes to unveil potential involved pathways of tissue-relevance.

### Training and validation of sarcoidosis classifier

To build a diagnostic model for sarcoidosis across tissues, the datasets were further divided into training and testing sets, each containing expression profiles from both blood and lung-associated tissue. Datasets GSE19314, GSE18781, GSE42834, GSE83456, GSE16538, GSE73394, and GSE148036 were used as training set to build the prediction model, while datasets GSE37912, GSE34608, GSE75023, GSE105149, GSE32887, and GSE119136 were included as testing sets. Candidate predictors were filtered by the criteria that the gene should be significantly differentially expressed in more than 3 of the 4 blood sets and in more than 2 of the 3 lung-tissue-associated sets. And those candidates with consistent regulatory directions across the discovery sets will be selected as predictors. The gene with log-transformed fold change (logFC) > 0 is regarded as a positive regulatory factor, and that less than 0 is a negative regulator. Classifier was generated by random forest (RF), Lasso and Elastic-Net Regularized Generalized Linear Models (GLMNET), and Gradient Boosting Machine (GBM) implemented in R package caret. The models were tuned using 10-fold cross-validation. Predictor selection and model training were performed only in training sets, and thus the other test sets could be used as external validation sets. The performance of classifiers was measured using threshold-dependent (sensitivity, specificity, accuracy) and threshold-independent ROC analysis (AUC). The prediction model with the highest performance in the training sets was chosen for assessment of predictive power in six independent testing datasets. To address the problem of systemic difference between cases and controls, randomly selected genes of identical size were compared to ensure the prediction power of model.

## Results

### Differential expression analysis and meta-signature identification

**FIGURE 2:**
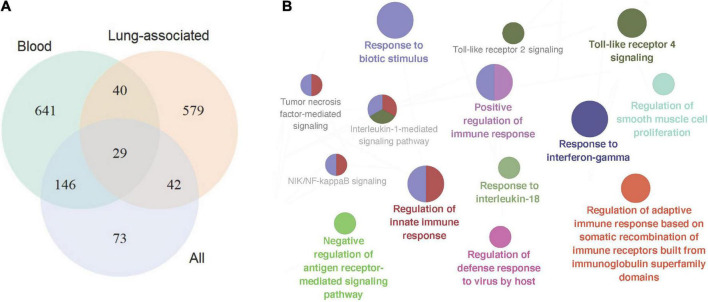
Signature gene identification in blood, lung-associated tissues, and all available tissue types. **(A)** Venn plot depicted differentially expressed genes in the three sets of meta-analyses, in peripheral blood samples, lung-associated tissues, and in all 13 cross-tissue samples combined, respectively. **(B)** Pathway enrichment analysis of the top signature genes in the meta-analysis across various tissues. The size of the nodes reflects the enrichment significance of the terms, and the colors denote groups of biological process.

In total 317 sarcoidosis patients and 339 healthy controls from the 13 GEO datasets were included in the final meta-analysis (Table 1 and Supplementary Table 1). Sarcoidosis patients included cutaneous, pulmonary, and lacrimal gland involvement. Random-effect models were applied to the 6 blood sets, the 4 lung-associated sets, and all 13 sets combined, respectively, to identify genes associated with sarcoidosis within blood, within lung, and between various tissues. Of the 12,968 genes available in at least 3 blood datasets and 3 lung tissue associated sets, 856 were significant at FDR < 0.05 in meta-analysis of peripheral blood, and 690 were significant in lung associated tissues, while 290 genes were differentially expressed when all 13 datasets from various tissues were combined in the meta-analysis (Figure 2A). Despite elevated significance and robustness of the meta-analysis, only 69 DEGs are commonly observed to be differentially expressed in blood and lung tissue, and 29 of them remain significant when more heterogenous sarcoid tissue are included (Table 2).

**TABLE 1:** Datasets used for cross-tissue meta-analysis and sarcoidosis classifier development.

GEO dataset	Platform	Tissue	Control samples	Sarcoid samples	ID
GSE19314	Affy U133 Plus 2.0 Array	Blood	20	38	Train1
GSE18781	Affy U133 Plus 2.0 Array	Blood	25	12	Train2
GSE42834	Illumina beadchip	Blood	113	61	Train3
GSE83456	Illumina beadchip	Blood	61	49	Train4
GSE16538	Affy U133 Plus 2.0 Array	Lung	6	6	Train5
GSE73394	Affy Gene 1.0 ST array	BAL	20	26	Train6
GSE148036	Illumina HiSeq 3000	Lung	5	5	Train7
GSE37912	Affy Exon 1.0 ST array	Blood	35	39	Test1
GSE34608	Agilent microarray	Blood	18	18	Test2
GSE75023	Affy U133A 2.0 array	AM	12	15	Test3
GSE105149	Affy U133 plus 2.0 array	Lacrimal gland	7	8	Test4
GSE32887	Affy U133 plus 2.0 array	Skin	5	26	Test5
GSE119136	Affy gene 1.0 ST array	Nasal brushing	12	14	Test6

Affy, Affymetrix Human Genome; AM, alveolar macrophage; Illumina beadchip, Illumina HumanHT-12 V4.0 expression beadchip; Agilent Microarray, Agilent-014850 Whole Human Genome Microarray 4 × 44K G4112F.

**TABLE 2:** Triple-significant differentially expressed genes (DEGs) in cross-tissue, blood, and lung-associated tissue meta-analyses.

Gene	Cross-tissue		Blood		Lung		Significance label*
			
	logFC	P-adjust	logFC	P-adjust	logFC	P-adjust	
*LINC01278*	−0.38	3.11 × 10^–13^	−0.35	8.72 × 10^–06^	−0.32	2.65 × 10^–03^	//++/?/??/++?
*GBP5*	1.55	5.56 × 10^–07^	1.47	4.58 × 10^–03^	1.57	3.21 × 10^–07^	+++++++/+?+//
*PSMB9*	0.55	1.11 × 10^–06^	0.45	3.80 × 10^–03^	0.56	3.18 × 10^–15^	++++++//++++/
*TAP2*	0.60	1.35 × 10^–06^	0.44	1.20 × 10^–03^	0.74	8.43 × 10^–06^	//++++//++++/
*PSTPIP2*	0.86	2.11 × 10^–06^	0.78	1.31 × 10^–02^	1.14	9.65 × 10^–22^	++++/±+++//
*CCNB1*	0.20	2.39 × 10^–06^	0.17	1.35 × 10^–05^	0.28	1.75 × 10^–03^	/////+/+//+//
*HDAC4*	0.13	1.69 × 10^–05^	0.16	6.52 × 10^–03^	0.13	1.90 × 10^–02^	//+//+///////
*SQOR*	0.48	5.99 × 10^–05^	0.54	2.11 × 10^–05^	0.34	5.85 × 10^–09^	++++/+??++++/
*FKBP14*	−0.22	8.74 × 10^–05^	−0.22	1.76 × 10^–02^	−0.24	8.38 × 10^–05^	/+///+//++///
*LINC00667*	−0.33	9.40 × 10^–05^	−0.37	1.96 × 10^–03^	−0.38	4.60 × 10^–03^	/+++/?/??///?
*CD38*	0.56	1.01 × 10^–04^	0.51	3.50 × 10^–02^	0.70	1.48 × 10^–02^	//++/+//+++//
*TAP1*	0.78	1.13 × 10^–04^	0.54	1.50 × 10^–02^	0.96	1.77 × 10^–05^	+++++?//++++?
*IFITM1*	0.49	1.77 × 10^–04^	0.38	1.25 × 10^–02^	0.77	1.80 × 10^–03^	//++/+/++++//
*PSME2*	0.42	2.67 × 10^–04^	0.46	7.30 × 10^–03^	0.31	8.73 × 10^–09^	/+++++///+++/
*THOC5*	0.13	3.09 × 10^–04^	0.16	3.60 × 10^–03^	0.14	5.61 × 10^–04^	///+/+///+///
*ANKRD22*	1.45	5.61 × 10^–04^	1.72	2.36 × 10^–03^	1.44	1.63 × 10^–04^	+++++++/+?+//
*CXCL10*	1.24	5.61 × 10^–04^	0.99	1.79 × 10^–02^	1.34	1.17 × 10^–06^	+++++++/+++//
*IDO1*	1.09	6.24 × 10^–04^	1.14	1.52 × 10^–02^	0.93	3.24 × 10^–02^	/+++++//+++//
*KCNJ2*	0.64	9.30 × 10^–04^	0.68	7.96 × 10^–03^	0.79	7.32 × 10^–03^	+++++///+++//
*CD300A*	0.41	1.06 × 10^–03^	0.26	4.99 × 10^–02^	0.59	2.01 × 10^–03^	/+++/++/+++//
*ETV7*	0.55	1.35 × 10^–03^	0.79	8.78 × 10^–03^	0.25	1.44 × 10^–06^	++++/+//++/+/
*BTN3A2*	0.35	1.58 × 10^–03^	0.20	1.61 × 10^–02^	0.47	2.89 × 10^–07^	//+/++///++//
*BLOC1S1*	0.20	1.85 × 10^–03^	0.25	3.72 × 10^–02^	0.17	2.79 × 10^–02^	//+/////+////
*RAB32*	0.25	2.58 × 10^–03^	0.26	1.67 × 10^–02^	0.15	3.22 × 10^–02^	//++////+/++/
*KIF1B*	0.19	7.84 × 10^–03^	0.31	4.11 × 10^–14^	0.17	2.10 × 10^–02^	//+//+//+////
*TPX2*	0.22	1.49 × 10^–02^	0.14	2.82 × 10^–02^	0.34	2.73 × 10^–02^	//++?/////+//
*RNASE6*	0.57	1.61 × 10^–02^	0.26	2.82 × 10^–02^	0.97	8.30 × 10^–04^	//++/+//+++//
*TSEN2*	−0.29	1.70 × 10^–02^	−0.39	1.94 × 10^–02^	−0.36	1.14 × 10^–02^	+++++//-++///
*ENTPD1*	0.35	2.69 × 10^–02^	0.36	1.83 × 10^–08^	0.46	1.44 × 10^–02^	+/+++//??++//

LogFC, log-transformed fold change; P-adjust, FDR-adjusted P-value; *: label of significance in each of the 13 GEO dataset, consistent with the order listed in Table 1.+: P-value < 0.05, fold change direction consistent with cross-tissue meta-analysis; −: P-value < 0.05, fold change direction contradictory with cross-tissue meta-analysis;/: P-value ≥ 0.05; ?: not available.

Top up-regulated genes in sarcoidosis include interferon signature genes such as *GBP5* and *IFITM1*, indicating active regulation of the interferon signaling. Chemokine genes induced by interferon such as *CXCL10* are also upregulated. Intriguingly, we identified genes significantly associated with sarcoidosis that caught limited attention in previous studies. These genes include the long non-coding gene *LINC01278* and some other genes known to be involved in the interferon network but lacking understanding of their role in pulmonary diseases, such as *IDO1* and *BTN3A2*.

Down-regulated genes that achieved significance in the meta-analysis of blood but failed in lung-associated tissues include *TRABD2A* and *NLRC3*. The former encodes for a metalloprotease and negative regulator or Wnt signaling, while the latter is characterized as a negative regulator of the type I IFN pathway. Up-regulated blood-specific genes include *MYD88*. The gene ranking top in lung-specific DEGs is the lncRNA gene *HCP5* and *IL10RA*. Most of the genes lack sufficient exploration as to their roles in pulmonary sarcoidosis, but more or less are associated with inflammatory pathways potentially of influence in the pathogenesis of the disease.

### Pathway enrichment analysis of sarcoidosis signature genes

Pathway enrichment analysis of the top 200 significant genes in meta-analysis revealed particular pathways that may be associated with sarcoidosis in blood and sarcoid tissues. 55 significant biological processes are functionally grouped into 10 critical pathways as shown in Figure 2B, In addition to the well-known IFN-γ response, we found activation of the cytokines IL-1 and IL-18. Host defense response to biotic stimuli, especially virus infection, is also significantly involved.

Top DEGs in meta-analysis of lung-associated tissue unveiled 16 groups of 36 pathways, most of them also observed in cross-tissue analysis (Supplementary Table 2). Although sarcoidosis is widely recognized as a Th1 disease, NK cells may also play a role in the pathogenesis. The biological process of cell-cell adhesion mediated by integrin is also significantly enriched in lung-associated tissues. With deepened understanding of integrin functions such as roles in cell survival, migration, and proliferation, potentials as therapeutic targets especially in respiratory disease may be exhibited. Pathway enrichment analysis in top genes of peripheral blood showed limited results with 18 biological processes divided into 4 groups, including mitosis, mRNA stabilization, IFN-γ signaling, and viral infection response (Supplementary Table 3). Unsurprisingly, interferon-gamma-mediated signaling pathways are consistently involved in all three meta-analyses.

### Biological processes enriched in tissue-specific differentially expressed genes in blood and lung

**FIGURE 3:**
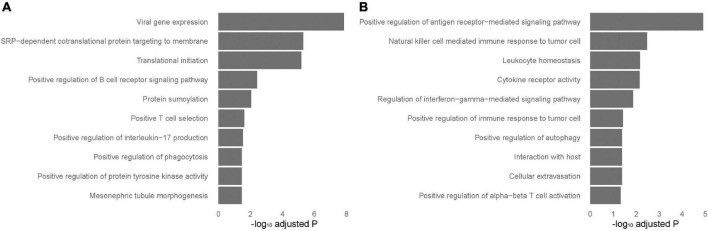
Tissue-specific pathways enriched in blood and lung-associated tissues. Enriched pathways specific to peripheral blood and lung-associated tissues. The analysis was performed based on the top differentially expressed genes specific to peripheral blood **(A)** and lung-associated tissues **(B)** identified in the results of meta-analyses.

To identify tissue-specific sarcoidosis signature in peripheral blood and sarcoid lung tissue, we further performed pathway enrichment based on the top 200 genes ranked by *P*-value selected from those DEGs that achieved significance in blood but not in lung-associated tissues or vice versa (Figure 3). Positive regulation of interleukin-17 (IL-17) production was significantly involved in blood-specific DEGs, concordant with previous understanding of this critical signaling pathway. Activation of autophagy was significantly enriched in both blood and lung-specific genes. The gene AIM2, functioning as a key factor of pyroptosis, was among the identified signatures for sarcoidosis, further emphasizing the role of cell death in sarcoidosis. NK cell mediated immune response to tumor cells was evident in DEGs in lung-associated tissues.

### Predictor selection and classifier establishment

**FIGURE 4:**
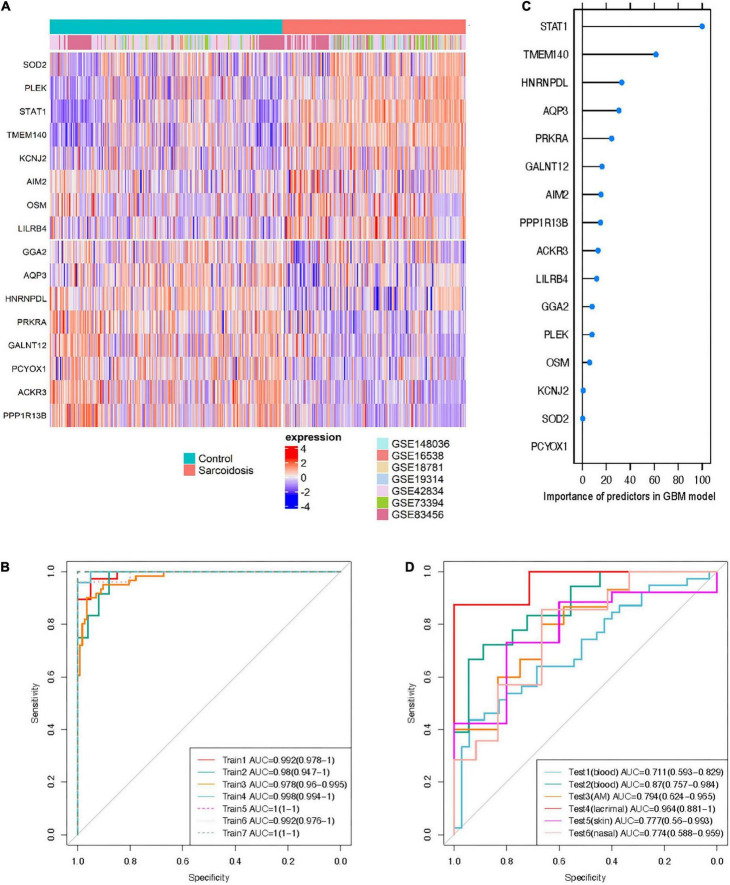
Cross-tissue classification model for sarcoidosis. **(A)** Heatmap shows expression profile of the 16 predictors in training sets. Half of the predictors were up-regulated while the others were down-regulated. **(B)** Importance of the predictors in GBM model. ROC curve of the classifier was shown in training sets **(C)**, and test sets **(D)**.

Sixteen genes met the requirement in the training sets based on the variable selection criteria with consistent directions of fold change across all discovery sets (*n* = 447), indicating robust dysregulation across datasets and thus were used for training of classifier (Figure 4A). All of these 16 genes function as protein-coding genes, and 8 were up-regulated while the other half were down-regulated. GBM and RF outperformed GLMNET in respect of both AUC and accuracy (GBM: AUC = 0.985, Accuracy = 0.937; RF: AUC = 0.998, Accuracy = 0.978; GLMNET: AUC = 0.949, Accuracy = 0.895) (Figure 4B and Supplementary Figure 1). The importance of each gene as a predictor variable was evaluated in the model (Figure 4C). *STAT1* provides the heaviest weight in all three models amongst the 16 predictors. Other predictors making major contributions include *TMEM140*, *AQP3*, and *SOD2*.

The independent performance of GBM and RF classifiers was evaluated in the 6 external testing sets (*n* = 209). The GBM model performed more reliable than the RF model in both blood and sarcoid tissues (AUC 0.711–0.964), possibly owing to problem of over-fitting in RF model despite cross-validation (Figure 4D and Supplementary Table 4). The lacrimal tissue set achieved the best AUC with 0.964 (accuracy = 0.933). We also randomly selected gene sets of identical size from the candidates that were available across all 13 datasets. With identical training and testing parameters, the model built from 16 randomly selected genes achieved an AUC of 0.464, 0.596, and 0.528 using the algorithms of GLMNET, GBM, and RF, respectively (Supplementary Figure 1), further emphasizing that the performance of the 16-gene set can be attributed to the biological significance of these consistently differentiated genes.

## Discussion

A meta-analysis of transcriptome-wide association studies was conducted between sarcoidosis and normal subjects across tissues by means of mining public datasets. We identified robust and novel genes potentially associated with sarcoidosis. A disease prediction classifier was subsequently built using machine learning and validated in independent datasets to discover candidate cross-tissue sarcoidosis biomarkers.

The expression profile of 29 genes was significantly associated with sarcoidosis the in meta-analysis of blood, lung, and cross-tissue datasets. Intriguingly, the long intergenic non-protein coding RNA *LINC01278* was found to be down-regulated in sarcoid tissues. *LINC01278* has been proposed to negatively regulate accumulation of β-catenin and ultimately inhibit the transcription of downstream target genes activated by Wnt/β-catenin signaling (23). Evidence of increased pulmonary Wnt-activation has been reported in sarcoidosis, potentially regulating myofibroblast differentiation of lung resident mesenchymal stem cells (24). Although *LINC01278* had never been observed to play an explicit role in sarcoidosis, it might contribute to the disease in critical biological pathways.

The role of the MHC genes in presenting antigen and triggering activation of T cells makes them good candidates for involvement in sarcoidosis. *PSMB9*, a gene downstream of *STAT1*, which is known to integrate with *IFNG* and to play a proteolytic role in MHC1 antigen presentation, has been reported to be upregulated in sarcoidosis (25). Non-MHC genes, *TAP1*, and *TAP2*, encoding the transporter associated with antigen processing, which participate in the antigen processing pathways prior to its presentation, are also interesting candidates and have been observed to be upregulated in sarcoidosis. A polymorphism of *TAP2* detected in patients with sarcoidosis further validated this point (26).

Multifunctional membrane surface glycoprotein (*CD38*) is considered as a marker of immune activation and involved in the regulation of lymphocyte adhesion to endothelial cells. Both CD3^+^CD4^+^CD38^+^ and CD38+ B cell subsets were found to be elevated in BAL as markers of an acute immune response in sarcoidosis patients (27, 28). In addition to lymphocytes, *PSTPIP2*, a gene supposed to be associated with autoinflammatory processes of macrophages in a mouse model, was found to be upregulated in progressive fibrotic pulmonary sarcoidosis (12).

Aberrant HDAC enzyme activities are evident in fibrotic diseases, of which *HDAC4* is important in lung fibrosis by modulating the production of ECM in lung myofibroblasts (29). Although widely accepted as a key factor in IPF, no HDAC inhibitors (HDACIs) have been investigated in sarcoidosis. Some dysregulated genes discovered in our meta-analysis have not been found to be associated with sarcoidosis previously, but variations of these genes such as *CCNB1*, *BLOC1S1*, and *KIF1B* are associated to some extent with fibrotic diseases, including complication of sarcoidosis and tuberculosis.

Enrichment of biological processes was performed in the top 200 genes ranked by *P*-value in the meta-analysis of blood, lung, and all datasets, respectively. Biological regulation of NK cells and myeloid cells seem to play a role in sarcoidosis. Increased cells of NK lineage were observed in our single-cell dataset of BAL. It is known that a subpopulation of CD56+ NK cells is activated and produces IFN-γ and TNF-α in sarcoidosis patients, implying involvement of these cells in granuloma formation (30). A strong Th2-M2 polarization was identified in both pulmonary and muscular sarcoidosis (31). Biological processes enriched from blood-specific DEGs revealed positive regulation of IL-17 and tyrosine kinase activity, while both blood and lung-specific genes showed activation of autophagy. Lung-specific genes otherwise were enriched for response of tumor, especially mediated by NK cells. The occurrence of a sarcoid-like localized or distant granulomatous reaction in cancer has been widely realized. In fact, sarcoidosis can occur before, during, or after the onset of solid or hematological malignancies.

To ensure the independence between training and testing sets, predictors of the cross-tissue classifier were selected based on the 7 training sets, and thus moderately different from DEGs in our meta-analysis. Interestingly, *KCNJ2*, one of the identified cross-tissue signature genes, is included as a predictor, which is activated in IPF but lacks exploration in sarcoidosis (32).

In the past few years, multiple prediction models based on transcriptomic signature have been developed in order to assist in the diagnosis of sarcoidosis, but none of them is currently used in clinical management (13, 33, 34). Intriguingly, microRNAs are frequently used in such models and perform well in diagnosis. Current models are built based on gene signature in peripheral blood or PBMC. The linear signature score is the most commonly used method to build classification models by assigning weights to selected gene markers. Two 8-microRNA diagnostic models achieved accuracy of 0.86 and 1, respectively in development datasets, suggesting that microRNAs might act as a crucial regulator in the pathogenesis (33, 34). The other two models were built using 20 and 17 genes, while long non-coding RNA genes were repeatedly identified as predictors (13, 34). *STAT4* and other factors of interferon signaling, as well as cytokine-related genes like *IL6ST* are major gene markers. These two models also performed well with an accuracy of 0.86 and AUC of 0.87, respectively. Compared with these diagnostic models, our classifier is the first cross-tissue model to predict diagnosis of sarcoidosis, indicating a scheme of systematic transcriptomic alteration over the body. However, whether the diagnostic model based on transcriptome can get universally applied assisting clinical decision still need further studies to validate.

We have to acknowledge some limitations of this study. First, meta-analysis suffers from inherent statistical limitations since the datasets lack concordance being derived from different batches, techniques, and platforms, although surrogate variables were estimated in our study. Also, the definitions of sarcoidosis and controls are slightly different among the datasets, but largely consistent and broadly acceptable. Second, whereas sarcoidosis is a heterogenous and complicated disease, clinical characteristics and disease status were not provided in most public datasets, limiting the clinical interpretation of significant genes. Third, particularly differentially expressed genes were not validated biologically in our study. Further experiments are needed to identify exact role they play in sarcoidosis. Lastly, our classifier tested only sarcoidosis vs. “healthy” controls but not vs. other granulomatous or interstitial lung diseases. The diagnostic power of this classifier to discriminate sarcoidosis from other diseases remains to be investigated.

This transcriptomics-based meta-analysis identified gene expression profiles and shared pathways associated with sarcoidosis across various tissues. This allowed to construct a 16-gene diagnostic classifier for sarcoidosis that potentially can complement more invasive procedures. Its precise diagnostic power needs to be validated in more abundant datasets of various tissues also from patients with other diseases.

## Data availability statement

The datasets presented in this study can be found in online repositories. The names of the repository/repositories and accession number(s) can be found in the article/Supplementary material.

## Ethics statement

This was a meta-analysis using previously publicated data on public database. The used data were approved previously individually. The patients/participants provided their written informed consent to participate in this study.

## Author contributions

HD and CW contributed to conception and design of the study. YJ conducted the analysis. YJ, DJ, and HD drafted the manuscript. UC contributed to the revision of the manuscripts. All authors contributed to manuscript revision and approved the submitted version.

## Footnotes

1
http://www.ncbi.nlm.nih.gov/geo
