# Supplementary material for: A transcriptomics-based meta-analysis identifies a cross-tissue signature for sarcoidosis
Source: Front Med (Lausanne). 2022 Sep 20;9:960266. doi: 10.3389/fmed.2022.960266 (PMC9530451; doi:10.3389/fmed.2022.960266)
Supplement: Supplementary file 1 [file Data_Sheet_1.docx]

Supplementary Material

## Supplementary Tables

Supplementary Table 1. Demographic features of samples used for meta-analysis in each dataset

| GEO dataset | Tissue | Control samples | Sarcoid samples | ID | Gender  (Male%) | | Age  (mean, sd) | |
| --- | --- | --- | --- | --- | --- | --- | --- | --- |
|  |  |  |  |  | Control | Sarcoidosis | Control | Sarcoidosis |
| GSE19314 | Blood | 20 | 38 | Train1 | 25% | 44.7% | 51.7 (10.7) | 51.3 (10.7) |
| GSE18781 | Blood | 25 | 12 | Train2 | 23.5% | 61.9% | 46.9 (15.6) | 46.8 (15.7) |
| GSE42834 | Blood | 113 | 61 | Train3 | 36.3% | 44.3% | N/A | N/A |
| GSE83456 | Blood | 61 | 49 | Train4 | 54.1% | 59.2% | 35.8 (13.6) | 47.8 (13.0) |
| GSE16538 | Lung | 6 | 6 | Train5 | N/A | N/A | N/A | N/A |
| GSE73394 | BAL | 20 | 26 | Train6 | 60% | 65.4% | 31.8 (15.7) | 48.5 (16.7) |
| GSE148036 | Lung | 5 | 5 | Train7 | 60% | 40% | 48.4 (17.2) | 34.6 (7.3) |
| GSE37912 | Blood | 35 | 39 | Test1 | N/A | N/A | N/A | N/A |
| GSE34608 | Blood | 18 | 18 | Test2 | 33.3% | 33.3% | N/A | N/A |
| GSE75023 | AM | 12 | 15 | Test3 | N/A | N/A | N/A | N/A |
| GSE105149 | Lacrimal gland | 7 | 8 | Test4 | 52.9% | 25% | N/A | N/A |
| GSE32887 | Skin | 5 | 26 | Test5 | N/A | N/A | N/A | N/A |
| GSE119136 | Nasal brushing | 12 | 14 | Test6 | 50% | 64.3% | 51.6 (14.5) | 52.1 (14.2) |

-

# Supplementary Table 2. Pathways enriched in the top DEGs in meta-analysis of lung-associated tissues

| Biological process | FDR | Associated Genes Found |
| --- | --- | --- |
| Adaptive immune response | 2.15×10^-10^ | ADA, BTLA, BTN3A1, BTN3A2, CAMK4, CCR6, CD160, CD3D, CD3G, CD48, CXCL10, EOMES, GPR183, IFNG, IL18RAP, IRF1, LEF1, MCOLN2, NECTIN2, PIK3CD, PRDM1, PRKCB, SIT1, SKAP1, SLAMF1, SLAMF7, TAP1, TAP2, TARP, TFEB, TNF, TNFRSF1B, TRAT1 |
| T cell activation | 4.31×10^-07^ | ADA, BTLA, BTN3A1, CAMK4, CCR6, CD160, CD3D, CD3G, CD5, CORO1A, EOMES, GLI3, GPR18, GPR183, IFNG, IRF1, LEF1, PIK3CD, PRDM1, PREX1, SIT1, TARP, TNFRSF1B |
| T cell receptor signaling pathway | 1.35×10^-06^ | ADA, BTN3A1, BTN3A2, CD160, CD3D, CD3G, NECTIN2, PIK3CD, PSMB8, PSMB9, PSME1, PSME2, SKAP1, TARP, TRAT1 |
| Alpha-beta T cell activation | 5.25×10^-04^ | ADA, CD160, EOMES, GLI3, GPR18, GPR183, IFNG, IRF1, LEF1, PRDM1 |
| Regulation of innate immune response | 7.70×10^-04^ | CD160, GBP5, IFNG, IL18RAP, IRF1, JAK1, NECTIN2, NLRC5, PSMB8, PSMB9, PSME1, PSME2, SERPINB9, SOCS3 |
| NK cell mediated cytotoxicity | 4.71×10^-03^ | CD160, CORO1A, IL18RAP, NECTIN2, SERPINB9, SLAMF7 |
| Leukocyte homeostasis | 8.80×10^-03^ | ADA, BCL2L11, CORO1A, GPR183, PIK3CD, PMAIP1, SIT1 |
| Placenta development | 8.91×10^-03^ | ADA, ADAM19, CCDC134, CCNF, EOMES, LEF1, PRDM1, SOCS3, TFEB |
| Membrane protein ectodomain proteolysis | 8.97×10^-03^ | ADAM19, IFNG, PTPN3, TNF, TNFRSF1B |
| T cell migration | 9.61×10^-03^ | CCR6, CD200R1, CXCL10, GPR183, ITGB7, PIK3CD |
| Antigen processing and presentation of peptide antigen via MHC class I | 1.02×10^-02^ | NCF1, PSMB8, PSMB9, PSME1, PSME2, TAP1, TAP2 |
| Myeloid leukocyte differentiation | 1.19×10^-02^ | CAMK4, GPR183, IFNG, LBR, LEF1, PARP1, PIK3CD, SBNO2, THOC5, TNF |
| Regulation of response to interferon-gamma | 1.75×10^-02^ | IFNG, JAK1, NLRC5, SOCS3 |
| Regulation of cell-cell adhesion mediated by integrin | 1.81×10^-02^ | ADA, FERMT3, SKAP1 |
| Interferon-gamma production | 2.79×10^-02^ | BTN3A1, BTN3A2, CD160, EOMES, IL12RB2, IL18RAP, TNF |
| Embryonic digestive tract development | 4.66×10^-02^ | ADA, ALDH1A2, GLI3, TNF |

# Supplementary Table 3. Pathways enriched in the top DEGs of meta-analysis in blood

| Biological process | FDR | Associated Genes Found |
| --- | --- | --- |
| Adaptive immune response | 2.15×10^-10^ | ADA, BTLA, BTN3A1, BTN3A2, CAMK4, CCR6, CD160, CD3D, CD3G, CD48, CXCL10, EOMES, GPR183, IFNG, IL18RAP, IRF1, LEF1, MCOLN2, NECTIN2, PIK3CD, PRDM1, PRKCB, SIT1, SKAP1, SLAMF1, SLAMF7, TAP1, TAP2, TARP, TFEB, TNF, TNFRSF1B, TRAT1 |
| T cell activation | 4.31×10^-07^ | ADA, BTLA, BTN3A1, CAMK4, CCR6, CD160, CD3D, CD3G, CD5, CORO1A, EOMES, GLI3, GPR18, GPR183, IFNG, IRF1, LEF1, PIK3CD, PRDM1, PREX1, SIT1, TARP, TNFRSF1B |
| T cell receptor signaling pathway | 1.35×10^-06^ | ADA, BTN3A1, BTN3A2, CD160, CD3D, CD3G, NECTIN2, PIK3CD, PSMB8, PSMB9, PSME1, PSME2, SKAP1, TARP, TRAT1 |
| Alpha-beta T cell activation | 5.25×10^-04^ | ADA, CD160, EOMES, GLI3, GPR18, GPR183, IFNG, IRF1, LEF1, PRDM1 |
| Regulation of innate immune response | 7.70×10^-04^ | CD160, GBP5, IFNG, IL18RAP, IRF1, JAK1, NECTIN2, NLRC5, PSMB8, PSMB9, PSME1, PSME2, SERPINB9, SOCS3 |
| Natural killer cell mediated cytotoxicity | 4.71×10^-03^ | CD160, CORO1A, IL18RAP, NECTIN2, SERPINB9, SLAMF7 |
| Leukocyte homeostasis | 8.80×10^-03^ | ADA, BCL2L11, CORO1A, GPR183, PIK3CD, PMAIP1, SIT1 |
| Placenta development | 8.91×10^-03^ | ADA, ADAM19, CCDC134, CCNF, EOMES, LEF1, PRDM1, SOCS3, TFEB |
| Membrane protein ectodomain proteolysis | 8.97×10^-03^ | ADAM19, IFNG, PTPN3, TNF, TNFRSF1B |
| T cell migration | 9.61×10^-03^ | CCR6, CD200R1, CXCL10, GPR183, ITGB7, PIK3CD |
| Antigen processing and presentation of peptide antigen via MHC class I | 1.02×10^-02^ | NCF1, PSMB8, PSMB9, PSME1, PSME2, TAP1, TAP2 |
| Myeloid leukocyte differentiation | 1.19×10^-02^ | CAMK4, GPR183, IFNG, LBR, LEF1, PARP1, PIK3CD, SBNO2, THOC5, TNF |
| Regulation of response to interferon-gamma | 1.75×10^-02^ | IFNG, JAK1, NLRC5, SOCS3 |
| Regulation of cell-cell adhesion mediated by integrin | 1.81×10^-02^ | ADA, FERMT3, SKAP1 |
| Interferon-gamma production | 2.79×10^-02^ | BTN3A1, BTN3A2, CD160, EOMES, IL12RB2, IL18RAP, TNF |
| Embryonic digestive tract development | 4.66×10^-02^ | ADA, ALDH1A2, GLI3, TNF |

# Supplementary Table 4. Performance of the cross-tissue diagnostic model in testing sets

| Testing set | AUC | accuracy | sensitivity | specificity | ppv | npv |
| --- | --- | --- | --- | --- | --- | --- |
| GSE37912 | 0.711 (0.593-0. 829) | 0.608 | 0.641 | 0.571 | 0.625 | 0.588 |
| GSE34608 | 0.870 (0.757-0.984) | 0.778 | 0.722 | 0.833 | 0.813 | 0.750 |
| GSE75023 | 0.794 (0.624-0.965) | 0.704 | 0.600 | 0.833 | 0.818 | 0.625 |
| GSE105149 | 0.964 (0.881-1) | 0.933 | 0.875 | 1.000 | 1.000 | 0.875 |
| GSE32887 | 0.777 (0.56-0.993) | 0.677 | 0.654 | 0.800 | 0.944 | 0.308 |
| GSE119136 | 0.774 (0.588-0.692) | 0.692 | 0.714 | 0.667 | 0.714 | 0.667 |

## Supplementary Figures

**Supplementary Figure 1.** Cross-tissue diagnostic classifier for sarcoidosis. (A) Performance of the cross-tissue classifiers for sarcoidosis built by GBM, RF, and GLMNET in training sets. (B) ROC of the model using randomly-selected variables as predictors.
